# A Newfoundland cohort of familial and sporadic idiopathic pulmonary fibrosis patients: clinical and genetic features

**DOI:** 10.1186/1465-9921-13-64

**Published:** 2012-08-01

**Authors:** Bridget A Fernandez, George Fox, Rick Bhatia, Eric Sala, Barbara Noble, Nash Denic, Dzintra Fernandez, Nigel Duguid, Amanda Dohey, Fady Kamel, Laura Edwards, Krista Mahoney, Susan Stuckless, Patrick S Parfrey, Michael O Woods

**Affiliations:** 1 Discipline of Genetics, Memorial University of Newfoundland, St John’s, NL, Canada; 2 Discipline of Medicine, Memorial University of Newfoundland, St John’s, NL, Canada; 3 Discipline of Radiology, Memorial University of Newfoundland, St John’s, NL, Canada; 4 Discipline of Pathology, Memorial University of Newfoundland, St John’s, NL, Canada; 5 Clinical Epidemiology Unit, Memorial University of Newfoundland, St John’s, NL, Canada

**Keywords:** TERT, Familial pulmonary fibrosis, Interstitial pneumonia, Telomere, Surfactant

## Abstract

**Background:**

Idiopathic pulmonary fibrosis (IPF) is an adult-onset Idiopathic Interstitial Pneumonia (IIP) usually diagnosed between age 50 to 70 years. Individuals with Familial Pulmonary Fibrosis (FPF) have at least one affected first or second-degree relative and account for 0.5-20% of cases.

**Methods:**

We ascertained and collected DNA samples from a large population-based cohort of IPF patients from Newfoundland, Canada. For each proband, a family history was documented and medical records were reviewed. Each proband was classified as familial (28 patients) or sporadic (50 patients) and all 78 probands were screened for variants in four highly penetrant, adult-onset PF genes (*SFTPC*, *SFTPA2, TERT,TERC*).

**Results:**

Seventy-eight IPF probands were enrolled of whom 28 (35.9%) had a positive family history. These 28 familial patients led to the recruitment of an additional 49 affected relatives (total of 77 FPF patients). By age 60 years, 42% of the familial cohort had been diagnosed with PF compared with only 16% of the sporadic patient collection (χ^2^ = 8.77, p = 0.003). Mean age of diagnosis in the familial group was significantly younger than the sporadic group (61.4 years vs. 66.6 yrs, p = 0.012) with a wider age range of diagnosis (19–92 years compared with 47–82 years). Thirty-three of 77 (42.8%) FPF patients had a tissue diagnosis and all but five had usual interstitial pneumonia histology. Compared with other published case series, the familial IIP histologies were more homogeneous. Three of 28 familial probands (10.7%) and none of the 50 sporadic probands had pathogenic variants in the four genes tested. All three familial probands had mutations in *TERT*. Other phenotypes associated with telomerase deficiency were present in these families including cirrhosis, bone marrow hypoplasia and premature graying. Telomere length assays were performed on mutation carriers from two families and confirmed telomere-related deficiency.

**Conclusion:**

The proportion of familial cases in our cohort is higher than any previously reported estimate and we suggest that this is due to the fact that Newfoundland cohort is ethnically homogeneous and drawn from a founder population. In our patient collection, diagnosis with IPF prior to age 45 years predicted familial disease. In two of the three *TERT* mutation families, the pedigree appearance is consistent with genetic anticipation. In the other 25 FPF families negative for mutations in known PF genes, we did not identify other telomerase associated medical problems (bone marrow dysfunction, cirrhosis) and we hypothesize that there are novel PF genes segregating in our population.

## Background

Idiopathic Pulmonary Fibrosis (IPF) is an adult-onset lung disease, which is a subtype of Idiopathic Interstitial Pneumonia (IIP). It is usually diagnosed between age 50 and 70 years and its histologic correlate is Usual Interstitial Pneumonitis (UIP). The only treatment that improves survival is lung transplantation. Without treatment, mean survival ranges from 2 to 4 years [1-3]. The prevalence of IPF in the United States is 14 per 100,000 [4].

Sporadic and familial forms of IPF have been recognized [5-7]. Risk factors for sporadic disease include male sex, cigarette smoking, metal and wood dust exposure and exposure to particular medications, including methotrexate and bleomycin [2,7].

Reports of familial forms of this disease (familial pulmonary fibrosis or FPF) date back to 1958 [8] and Online Mendelian Inheritance in Man lists FPF as an autosomal dominant disorder with variable penetrance (OMIM# 178500). Using a survey of respirologists in the United Kingdom, Marshall and colleagues [6] estimated the prevalence of FPF as 0.5-2.2% of all IPF cases. Using a similar survey of Finnish pulmonary clinics, 3.3-3.7% of Finnish cases were familial [9]; however, these studies may have underestimated the true proportion of PF patients who have an affected first- or second-degree relative. For example, 9/47 (19.1%) PF patients who received a lung transplant through the Vanderbilt lung transplant service reported a relative with Interstitial Lung Disease (ILD) [10] and in a review of the genetics of PF, these authors indicated that, in their experience, 20% of PF is familial [11]. In a Netherlands IPF cohort of 118 unrelated patients, 19.5% were classified as having FPF with 2 or more affected first-degree relatives [12].

Several publications have compared the clinical features of familial and sporadic PF patients, and there were no distinguishing features apart from a younger mean age at diagnosis (ranging from 3.5 to 12 years) in the familial compared with the sporadic groups [6,7,9,12,13].

Eight to 18% of FPF patients have autosomal dominant mutations in one of the two genes encoding the essential components of telomerase: *TERT* (telomerase reverse transcriptase) and *TERC* (telomerase RNA component) [14]. Telomerase maintains the integrity of the ends of chromosomes and all mutation-positive individuals have telomere shortening of circulating lymphocytes [15-17]. Alder et al. [18] screened 100 sporadic PF patients and found that 10% had telomere lengths < 1^st^ percentile for age, and Cronkhite et al. [17] found that telomere length was less than the 10^th^ percentile in 23% of sporadic and 24% of familial PF cases. This suggests that in at least one-quarter of PF patients, perturbations in telomere maintenance are involved in disease pathogenesis.

Autosomal dominant mutations in surfactant protein C (*SFTPC*) and in surfactant protein A2 (*SFTPA2*) have also been indentified in familial IIP patients, but these genes appear to be infrequently mutated in familial and sporadic cohorts [19-21], with the exception of the Dutch cohort reported in 2010 [12].

In this study we have addressed the following research questions: (1) What is the prevalence of pulmonary fibrosis in Newfoundland? (2) Are there significant clinical differences between sporadic and familial PF?, and (3) What is the contribution of the known, highly penetrant pulmonary fibrosis causing genes in this population?

## Methods

### Proband and family recruitment

From January 2006 to July 2011**,** we attempted to ascertain all prevalent cases of IPF/FPF in the Canadian province of Newfoundland and Labrador (NL), which has a population of 509,000 [22]. The province’s five respirologists referred any patient with a diagnosis of IPF to the study. Ninety percent of probands agreed to participate. For each consenting patient, medical records were reviewed. Probands either had biopsy proven UIP on a clinical pathology report or, in the absence of surgical lung biopsy, met the ATS/ERS (American Thoracic Society/European Respiratory Society) criteria for IPF [3], with the caveat that abnormal pulmonary function testing was required show evidence of restriction and/or impaired gas exchange. Memorial University’s Human Investigations Committee approved this study (#02.26) and all individuals consented to be in the study.

Each eligible proband completed a family history form, a medical history form and a questionnaire that included questions about tobacco use, occupation, and exposure to fibrogenic dusts and drugs. Patients with significant environmental exposures known to be associated with lung fibrosis, or with known collagen vascular diseases, were excluded from the study.

Using the family history, each proband was classified as sporadic, familial or equivocal. Sporadic probands had no family history of PF on a three-generation pedigree. A familial proband was defined as an individual with PF who had at least one affected first- or second-degree relative (with the diagnosis of PF in the relative confirmed by medical record review). Relatives of familial UIP probands who had other IIP histologies were also asked to participate. Within each family, we attempted to recruit all adult first-degree relatives of each affected family member. Relatives were made aware of the possibility of a subclinical diagnosis and those who consented completed the enrolment protocol including Pulmonary Function Tests (PFTs) and a high-resolution chest CT scan (HRCT). Probands with family histories suggestive of inherited lung disease, but whose affected relatives declined participation (so that chart review was not possible), were classified as having equivocal family histories.

### Radiologic, pathologic and clinical phenotyping

Two radiologists (RB, ES) independently reviewed the chest radiographs and HRCT scans of all participants (probands and relatives). Each HRCT was scored at five levels using the Royal Brompton Hospital system [23] and was classified into one of four categories: “definitely affected with IPF”, “probably affected”, “possibly affected” (early disease), and “unaffected”.

All pathology specimens (surgical lung biopsies and autopsied lungs) were reviewed by one of two pathologists (ND, DF). The specimens were classified as normal or abnormal. If abnormal, the histologic pattern was described. The radiologists and pathologists were blinded to each participant’s clinical status.

A clinical panel (BAF, GF, BN) reviewed each participant’s clinical history, PFTs and radiology (+/− pathology) designation. The panel gave each participant a final classification of “definitely affected” with IPF (tissue diagnosis of UIP and for familial cases, other IIP histology patterns were also accepted), “probably affected” (no surgical biopsy or autopsy, but characteristic HRCTs and PFTs with evidence of impaired gas exchange and/or restriction), “possibly affected” (PFTs or HRCTs abnormal and consistent with early ILD), or “unaffected”.

### Analysis of clinical data

Only participants classified as “definitely” or “probably affected” with IPF by the clinical panel were included in the following analyses*.* Clinical and physiologic data from the FPF group (77 patients) and the sporadic PF group (50 patients) were analyzed using descriptive statistics. The familial and sporadic groups were compared using standard statistical approaches (*χ*2 test, Student *t*-test with two-tailed distribution). For some analyses, the group of affected individuals with FPF was limited to those who were diagnosed because they developed respiratory complaints (57 patients), excluding (because of lead-time bias) those who were diagnosed through this study by clinical screening.

### DNA sequencing of candidate genes

Four of the known genes causing familial pulmonary fibrosis were sequenced in 28 FPF probands and 50 sporadic PF patients: *TERTTERCSFTPC* and *SFTPA2*. DNA was extracted from whole blood using either a simple salting out method [24] or by using the Wizard Genomic DNA Purification Kit (Promega Corporation, Madison, WI). DNA sequencing in both directions was performed for all exons and exon/intron boundaries. Primers and PCR conditions are available upon request. Primers were designed using the Primer3 v0.4.0 software application (http://frodo.wi.mit.edu/primer3/). Variant nomenclature and primers were derived from the following RefSeq accession numbers: NG_009265.1 (*TERT*), NG_016363.1 (*TERC*), NG_016968.1 (*SFTPC*) and NG_013046.1 (*SFTPA2*). Automated sequencing was performed on either an ABI 3130 Genetic Analyzer or an ABI 3730 Genetic Analyzer (Applied Biosystems, Foster City, CA).

### Analyses of telomere length

Five living mutation carriers from two of the three families with *TERT* mutations (R0851, R0892) had assays to determine telomere length in lymphocytes. Unfortunately, the only affected individual in family R1254 died before we could draw blood for the telomere assay. The procedure was performed by Repeat Diagnostics (Suite 209–267 West Esplanade, North Vancouver, BC, V7M1A5, Canada) to measure the length of telomeres in blood samples. Telomere lengths were then plotted on graphs indicating Caucasian population averages for telomere lengths in lymphocytes [25].

## Results

Clinical data: Seventy-eight unrelated IPF patients were enrolled, of whom 28 (35.9%) had a positive family history which was confirmed by record review and, where possible, by clinical assessment of the affected relatives. These 28 FPF probands led to the identification of an additional 49 relatives with PF. The largest families each contain eight affected individuals. With the exception of three families (R0851, R0892, R1254), all family histories were negative for other medical problems associated with telomerase mutations (e.g. unexplained bone marrow failure, cryptogenic cirrhosis) and there were no affected individuals with infantile or childhood onset ILD. The total FPF (n = 77) and sporadic groups (n = 50) were both slightly enriched for males (55.8% and 62%, respectively).

The R0896 family was initially ascertained as two separate families that were later genealogically linked. Also, six sporadic patients were reclassified as familial when another affected relative was referred to the study.

This IPF cohort is entirely Caucasian, predominantly of Irish/English ethnicity. Twenty-seven of the 28 familial probands and 47 of 50 sporadic patients described themselves as “Newfoundlanders” with Irish and/or English ancestors who have lived in the province for at least three generations. One familial proband was from Nova Scotia Canada (Scottish ethnicity). Two sporadic patients had parents who were born in the United Kingdom (English ancestory) and one of the parents of a third sporadic patient was born in Great Britain (English ancestory); his other parent was from Newfoundland.

As of July 30, 2011, the dataset contained 34 living familial and 19 living sporadic patients. Using an at risk population size of 400,925 (NL population > age 19) [22], the minimum prevalence of PF in Newfoundland is 13.22 per 100,000.

Fifty-seven of 77 (74.0%) FPF patients were diagnosed prior to enrolment in this study because they presented to physicians with respiratory symptoms (FPF-diagnosed clinically or FPF-DC). The remaining 20 FPF cases were diagnosed through this study by family screening (FPF-diagnosed screening or FPF-DS) (Table 1). The mean age of diagnosis was significantly different between the clinically diagnosed FPF group and the sporadic cohort (n = 50) (61.4 years versus 66.6 years, p = 0.012). Forty-two percent of the FPF patients who presented clinically were diagnosed by age 60 years, whereas only 16% of the sporadic patients were diagnosed by this age (χ^2^ = 8.77; p = 0.003). The cumulative risk of lung transplant and/or death was compared for the clinically diagnosed FPF patients and the sporadic patients. By age 60 years, 19% of the clinically diagnosed FPF patients were dead or had received a lung transplant compared with 2% of the sporadic group. However the mean age to death or transplant between the two groups was not statistically significant (FPF 68.9 years and sporadic 73.7 years; p = 0.466).

**Table 1:** Comparison of demographic and clinical variables between familial and sporadic PF patients

**Variables**	**Total FPF patients (N = 77)**	**FPF-DC (N = 57)**	**FPF-DS (N = 20)**	**Sporadic patients (N = 50)**	**Significance of difference**
Mean age symptom onset	57.11	57.97	N/A	63.85	**p = 0.012**
(years)					(FPF-DC vs. sporadic)
Mean age at diagnosis (years)	60.65	61.43	58.45	66.58	**p = 0.012**
					(FPF-DC vs. sporadic)
Mean age at death or	N/C	64.58 (n = 36)	73.62 (n = 6)	70.60 (n = 32)	**p = 0.025**
transplant (n = number of deceased or transplanted patients)					(FPF-DC vs. sporadic)
Gender					
Number (%) males	M = 43 (55.8 %)	M = 34 (59.6 %)	M = 9 (45.0 %)	M = 31 (62.0 %)	p = 0.531
Number (%) females	F = 34 (44.2 %)	F = 23 (40.4 %)	F = 11 (55.0 %)	F = 19 (38.0 %)	(total FPF vs. sporadic)
Current or ever smokers (%)	61/76 (80.3 %)	45/56 (80.4 %)	16/20 (80.0 %)	42/50 (84.0 %)	p = 0.767
					(total FPF vs. sporadic)
Number of patients with symptom at diagnosis:					FPF-DC vs. sporadic
Dyspnea	N/C	43 (75.4 %)	N/C	44 (88.0 %)	p = 0.096
Cough		37 (64.9 %)		35 (70.0 %)	p = 0.576
Chest pain		14 (24.6 %)		12 (24.0 %)	p = 0.946
Pneumonia		10 (17.5 %)		7 (14.0 %)	p = 0.617
Hemoptysis		5 (8.8 %)		1 (2.0 %)	p = 0.129
Pneumothorax		2 (3.5 %)		0 (0.0 %)	p = 0.181
Most specific diagnostic test:					
Number of patients (%)					
CXR	4 (5.2 %)	4 (7.0 %)	0 (0.0 %)	0 (0.0 %)	
HRCT	40 (51.9 %)	23 (40.4 %)	17 (85.0 %)	31 (62.0 %)	
Surgical lung biopsy	30 (39.0 %)	28 (49.1 %)	2 (10.0 %)	21 (42.0 %)	
Autopsy	3 (3.9 %)	2 (3.5 %)	1 (5.0 %)	0 (0.0 %)	
Treatments: Number of patients (%)					FPF-DC vs. sporadic
Prednisone	40 (51.9 %)	38 (66.7 %)	2 (10.0 %)	36 (72.0 %)	p = 0.551
Cyclophosphamide	7 (9.1 %)	7 (12.3 %)	0 (0.0 %)	2 (4.0 %)	p = 0.170
Azathioprine	12 (15.6 %)	11 (19.3 %)	1 (5.0 %)	12 (24.0 %)	p = 0.555
N-acetyl cysteine	16 (20.8 %)	13 (22.8 %)	3 (15.0 %)	20 (40.0 %)	p = 0.055
Lung transplant	5 (6.5 %)	5 (8.8 %)	0 (0.0 %)	3 (6.0 %)	p = 0.586

FPF = familial pulmonary fibrosis, FPF-DC = familial pulmonary fibrosis diagnosed because individual developed clinical symptoms of lung disease, FPF-DS = familial pulmonary fibrosis diagnosed because the individual had clinical screening based on family history, N/A = not applicable, N/C = not calculated, % = percentage, CXR = chest X-ray, HRCT = high resolution computerized tomography of chest.

Comparing the clinically diagnosed FPF and sporadic groups, there was no statistically significant difference in presenting symptoms or treatments. The commonest presenting symptom was dyspnea and the most frequently prescribed treatment was prednisone. The proportion of current or ever-smokers was similar between the two groups (FPF-diagnosed clinically 80.4%, sporadic PF 84.0%).

Thirty-three FPF patients had either a surgical lung biopsy or an autopsy, and of these 82% had UIP histology; only 5 family members had pathology classified as another form of IIP (Table 2). Twenty-one of the 50 (42%) sporadic patients enrolled had clinical pathology reports consistent with UIP. Following review by the research pathologist, four of these were reclassified as having another IIP histology and these cases were included in the subsequent analyses.

**Table 2:** Comparison of pulmonary function tests (PFTs) at diagnosis between clinically diagnosed FPF (FPF-DC), FPF diagnosed through screening (FPF-DS) and sporadic groups

**PFT parameter**	**Number (%) of FPF-DC patients with abnormal finding**^**a**^	**Mean % of predicted value +/− SD**	**Number (%) of FPF-DS patients with abnormal finding**^**a**^	**Mean % of predicted value +/− SD**	**Number (%) of sporadic PF patients with abnormal finding**^**a**^	**Mean % of predicted value +/− SD**
FVC	23/45 (51.1 %)	78.4 % +/− 15.4 %	6/16 (37.5 %)	92.7 % +/−16.8 %	24/46 (52.2 %)	80.1 % +/− 19.3 %
TLC	20/41 (48.7 %)	78.5 % +/− 15.4 %	6/16 (37.5 %)	86.6 +/−19.6 %	19/41 (46.3 %)	80.2 % +/− 17.9 %
FEV1	22/42 (52.3 %)	79.1 % +/− 15.1 %	5/16 (31.2 %)	87.1 % +/−12.9	21/46 (45.6 %)	81.5 % +/− 17.6 %
DL_CO_	39/40 (97.5 %)	56.4 % +/− 15.7 %	12/16 (75.0 %)	66.4 % +/−18.4	37/42 (88.0 %)	57.2 % +/− 23.2 %
Isolated reduction in DL_CO_ (other PFTs normal)	12/36 (33.3 %)	N/C	6/16 (37.5 %)	N/C	14/39 (35.9 %)	N/C

SD = standard deviation, FVC = forced vital capacity, TLC = total lung capacity, FEV1 = forced expiratory volume exhaled in the 1^st^ second, DL_CO_ = diffusing capacity of the lung for carbon monoxide.

^a^ Abnormal if: FVC, TLC, FEV1 or DL_CO_ < 80 % predicted.

The most consistently abnormal parameter on pulmonary function tests (PFTs; Table 2)**,** obtained at the time of diagnosis, was the Diffusing Capacity of the Lung for Carbon Monoxide (DL_CO_). It was below 80% of predicted in 97.5% and 88% of the FPF-diagnosed clinically and sporadic cases, respectively (respective mean DL_CO_s were 56.4% and 57.2% of predicted). Initial PFTs showed an isolated reduction of DL_CO_ in 33.3% and 35.9% in each of these respective groups. Because of our enrollment criteria, only about half of patients in each group showed initial evidence of restriction on PFTs.

### Sequencing of known PF-causing genes

Four genes previously known to cause hereditary pulmonary fibrosis (*TERTTERCSFTPC* and *SFTPA2)* were screened in our sporadic and familial cohorts, and the only mutations found were in *TERT* in three unrelated familial cases (Table 3). Hence we identified pathogenic variants in three of 28 familial probands (10.7%) and none of 50 sporadic probands. In family R0892, we identified a novel heterozygous *TERT* variant [c.2648 T > G (p.Phe883Cys)] which was found in all affected family members tested (n = 5) and is predicted to be “damaging” using SIFT and “probably damaging” using PolyPhen (Additional file 1: Table S1). In family R0851, we identified a novel putatively pathogenic *TERT* variant [c.1892 G > A (p.Arg631Gln)] that segregates in all affected members of the nuclear family (n = 4). When analyzed with bioinformatic programs, this variant was deemed “damaging” by SIFT, and “possibly damaging” by Polyphen. In family R1254, a heterozygous *TERT* mutation [c.2594 G > A (p.Arg865His)] was identified in the proband and his unaffected daughter (age 35). This variant has been previously published and proven to be pathogenic [15]. Numerous other variants were identified which we considered neutral (benign) or of unknown significance (Additional file 1: Table S1).

**Table 3:** Variants identified that are predicted to be deleterious

**Causative variant**	**Predicted protein change**	**Identified families**
*TERT*: c.2648 T > G	p.Phe883Cys	R0892
*TERT*: c.1892 G > A	p.Arg631Gln	R0851
*TERT*: c.2594 G > A	p.Arg865His	R1254

### Telomere length assays

**Figure 1:**
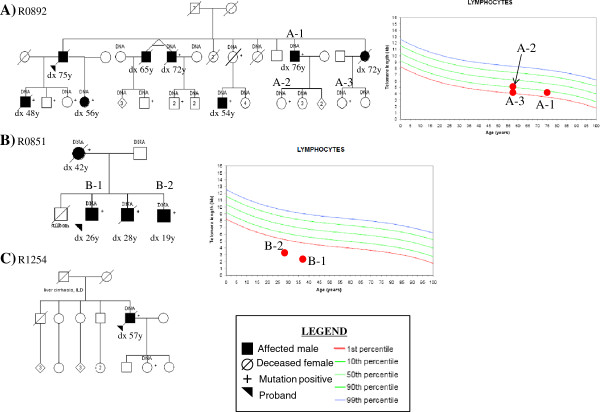
**Pedigrees of families with*TERT mutations.*** Mutation positive individuals are indicated by a “+” sign. Dx = Age at diagnosis of pulmonary fibrosis. A) Novel *TERT* variant: c.2648 T > G (p.Phe883Cys) segregates with the disease. B) Novel *TERT* variant: c.1892 G > A (p.Arg631Gln) segregates with disease. C) Previously reported pathogenic *TERT* mutation: c.2594 G > A (p.Arg865His) present in affected patient and unaffected daughter. ILD = Interstitial lung disease. Telomere assays were not performed in this family.

Telomere length assays carried out on five affected individuals with *TERT* mutations from two families (R0892 and R0851). All five patients had markedly short telomeres (≤10% ile of normal) when compared to Caucasian population age-matched averages (Figure 1), supporting the pathogenicity of the *TERT* variants identified in these two families.

## Discussion

Using a population based approach, we determined that the minimum prevalence of PF in NL is 13.2 per 100,000, comparable to the prevalence of 14–20 per 100,000 from more admixed populations [4,26]. However, the proportion of familial cases (approximately 36%) is higher than previously reported estimates that range from 0.5-20% [6,9,10,12]. Although this may be partly due to ascertainment bias (respirologists being more likely to refer familial than sporadic cases), the province has a relatively small number of respirologists and we were able to regularly communicate with them to ensure more complete recruitment of patients regardless of family history. Hence the Newfoundland founder population may be enriched for familial forms of PF. This Canadian island is composed of a series of genetic isolates and founder mutations have been demonstrated for several autosomal dominant disorders including arrhythmogenic right ventricular dysplasia type 5, Lynch syndrome and hereditary diffuse gastric cancer syndrome [27-29]. This coupled with large family sizes has led to increased prevalence of these genetic diseases in NL.

van Moorsel et al. (2002) [12] reported the next highest proportion of familial cases in a UIP cohort, 23 of 118 IPF probands or 20%. The patients were ascertained from a single Dutch ILD clinic, and although ethnicities were not specified, the Netherlands has a relatively homogeneous population [30]. Five of 20 Dutch IPF families segregated an *SFTPC* mutation [12], whereas no pathogenic variants in *SFTPC* were found in 73 Newfoundland probands. Although the five Dutch SFTPC cases had UIP histology, their HRCT scans were not typical of IPF, with features that included diffuse lung involvement, extensive ground glass and multiple cysts In contrast, our 28 familial probands, who all screened negative for *SFTPC* mutations, had HRCT scans which were classified by our blinded radiology panel as “definitely” or “probably” affected with IPF. The lower proportion of familial disease reported by other groups [6,9] is almost certainly due, at least in part, to the limitations of identifying familial cases by mailing questionnaires to patients and health care providers.

Consistent with previously published work [6,7,9,12], we found that individuals with familial disease had a younger mean age of diagnosis with no other clinically distinguishing features (Table 4). Mean diagnosis age in the Newfoundland FPF cohort (excluding relatives who were diagnosed through clinical screening) was only five years younger than the sporadic group, however the FPF group had a wider range of age at diagnosis (19–92 years compared with 47–82 years in the sporadic group). Fifteen percent of the Newfoundland FPF cohort was diagnosed by 40 years; no sporadic case was diagnosed before age 47. Hence in our patient collection the diagnosis of PF below age 45 predicted familial disease. Interestingly, in the only penetrance study reported to date, investigators reviewed 134 patients with *TERT* mutations. PF never occurred before age 40, and developed in 60% of male mutation carriers by age 60 [31].

**Table 4:** Idiopathic interstitial pneumonia (IIP) histologies among FPF and sporadic PF patients with a tissue confirmed diagnosis (surgical lung biopsy or autopsied lungs)

	**All FPF patients (n = 77)**	**Sporadic PF patients (n = 50)**
Number of patients with a tissue diagnosis (%)	33 (42.9 %)	21 (51 %)
Number of patients with IPF/UIP histology (%)	27 (81.8 %)	16 (76.2 %)
Number of patients with mixed UIP and NSIP histology patterns (%)	1^a^	1
Number with IIP other than UIP (%):	3	2
NSIP	0	0
COP	0	0
AIP	0	0
RB-ILD	0	0
DIP	0	0
LIP Unclassifiable interstitial pneumonia	2 ^a^	2
Total non-UIP histologic pattern	6 (18.2 %)	4 (19 %)

NSIP = non-specific interstitial pneumonia, COP = cryptogenic organizing pneumonia, AIP = acute interstitial pneumonia, RB-ILD = respiratory bronchiolitis-associated interstitial lung disease, DIP = desquamative interstitial pneumomia, LIP = lymphocytic interstitial pneumonia.

^a^ One biopsy showing a mix of UIP and NSIP and one biopsy showing unclassifiable interstitial pneumonia come from 2 members of the same family (R0892). The other 4 non-UIP biopsies from PF patients come from four different families.

In our IPF patient collection of 127 individuals, 54 patients (43%) had biopsy proven IIP, 81% of which was UIP (Table 2)**.** Those who did not have a biopsy were classified as having “probable IPF” if the HRCT showed typical bibasilar abnormalities and if PFTs showed evidence of restriction or impaired gas exchange or both. The majority of patients in both groups had a low DL_co_ at diagnosis (97.5% and 88% in the familial and sporadic groups respectively), but only half of the patients in each group showed initial evidence of restriction. Table 5 summarizes the most recent follow-up PFT data from the FPF and sporadic patients, showing that over time the proportion of patients with restriction rose to 69%and 62.5% in the clinically diagnosed FPF and sporadic groups.

**Table 5:** Comparison of pulmonary function tests (PFTs) obtained at most recent follow-up between clinically diagnosed FPF (FPF-DC), FPF diagnosed through screening (FPF-DS) and sporadic groups

**PFT parameter**	**Number (%) of FPF-DC patients with abnormal finding**^**a**^	**Mean % of predicted value +/− SD**	**Number (%) of FPF-DS patients with abnormal finding**^**a**^	**Mean % of predicted value +/− SD**	**Number (%) of sporadic PF patients with abnormal finding**^**a**^	**Mean % of predicted value +/− SD**
FVC	32/44 (72.7 %)	70.5 % +/−19.5 %	6/16 (37.5 %)	90.3 % +/−17.8 %	33/47 (70.2 %)	69.9 % +/− 18.6 %
TLC	20/29 (69.0 %)	69.9 % +/− 17.0 %	5/15 (33.3 %)	85.6 % +/−20.3 %	20/32 (62.5 %)	69.2 % +/− 17.2 %
FEV1	35/43 (81.3 %)	68.6 % +/− 18.6 %	7/16 (43.8 %)	83.2 % +/−15.6 %	32/47 (68.0 %)	71.8 % +/− 18.5 %
DL_CO_	34/35 (97.1 %)	47.6 % +/− 20.1 %	15/16 (93.8 %)	60.2 % +/−13.8 %	35/38 (92.1 %)	47.3 % +/− 26.4 %
Isolated reduction in DL_CO_ (other PFTs normal)	4/25 (16.0 %)	N/C	6/15 (40.0 %)	N/C	2/29 (6.9 %)	N/C

SD = standard deviation, FVC = forced vital capacity, TLC = total lung capacity, FEV1 = forced expiratory volume exhaled in the 1^st^ second, DL_CO_ = diffusing capacity of the lung for carbon monoxide.

^a^ Abnormal if: FVC, TLC, FEV1 or DL_CO_ < 80 % predicted.

Five earlier publications have described the clinical features of familial IPF or IIP cohorts [6,7,9,12,32] and in three of these comparisons were made to a sporadic PF group collected by the authors [7,9,12]. In these five studies, familial PF was defined as at least two cases of PF within the same biologic family [6,7,9] or at least 2 cases in first-degree relatives [12,32].

In 2000, Marshal et al. [6] identified 21 IIP families (containing 57 affected individuals) by mailing surveys to adult respirologists in the United Kingdom. The cohort was probably ethnically heterogeneous and no attempt was made to determine the patients’ IIP classification. Mean age of diagnosis was younger than in our familial cohort (55.5 years versus 61.4 years) and the UK cohort had a lower proportion of current or ever smokers (50% versus 80.3%).

In 2005 Lee et al. [7], published a well characterized collection of 15 familial IPF families containing 47 affected individuals, with detailed clinical information on 27 patents. This group represented all familial IPF patients treated at the Mayo clinic in Minnesota over a 10-year period. The 27 familial cases were compared to 63 sporadic IPF patients previously collected at their institution. All available biopsies showed UIP. The main familial versus sporadic comparisons were age at diagnosis (59.4 years versus 63 years), and median survival which was similar between the 2 groups, at 2–3 years.

Steele et al. (2005) [32] recruited the largest familial cohort to date, 111 families with IIP from three major U.S. ILD treatment centers, containing 309 patients “definitely” or “probably affected” with IIP. As observed in the 28 Newfoundland IPF families, the age range of diagnosis within American cohort was wide (30.3–95.4 years). Fifty of the 111 families (45%) had radiology or histopathology findings consistent with the presence of more than one form of IIP within the family. In our familial group, only 18.2% of the patients had non-UIP histology (Table 2). The unidentified PF genes segregating in the Newfoundland population may be associated with a more homogenous phenotype than is typical of the known PF causing genes. Alternatively, our recruitment protocol may have biased against ascertainment of families with multiple IIP histologies. While we accepted relatives with non-UIP histologies, all probands were required to have a clinical diagnosis of IPF/UIP.

van Moorsel et al. (2010) [12] compared 22 unrelated familial IPF patients from one Dutch centre to 95 sporadic IPF cases. Their familial group consisted of probands only, and their data confirmed that of the earlier studies. No familial versus sporadic differences were identified apart from younger diagnosis age in the familial group. Although not statistically significant, the Newfoundland familial cohort was less enriched for males than the sporadic patient collection (Table 4), and this was also observed in van Moorsel’s study [12].

**Figure 2:**
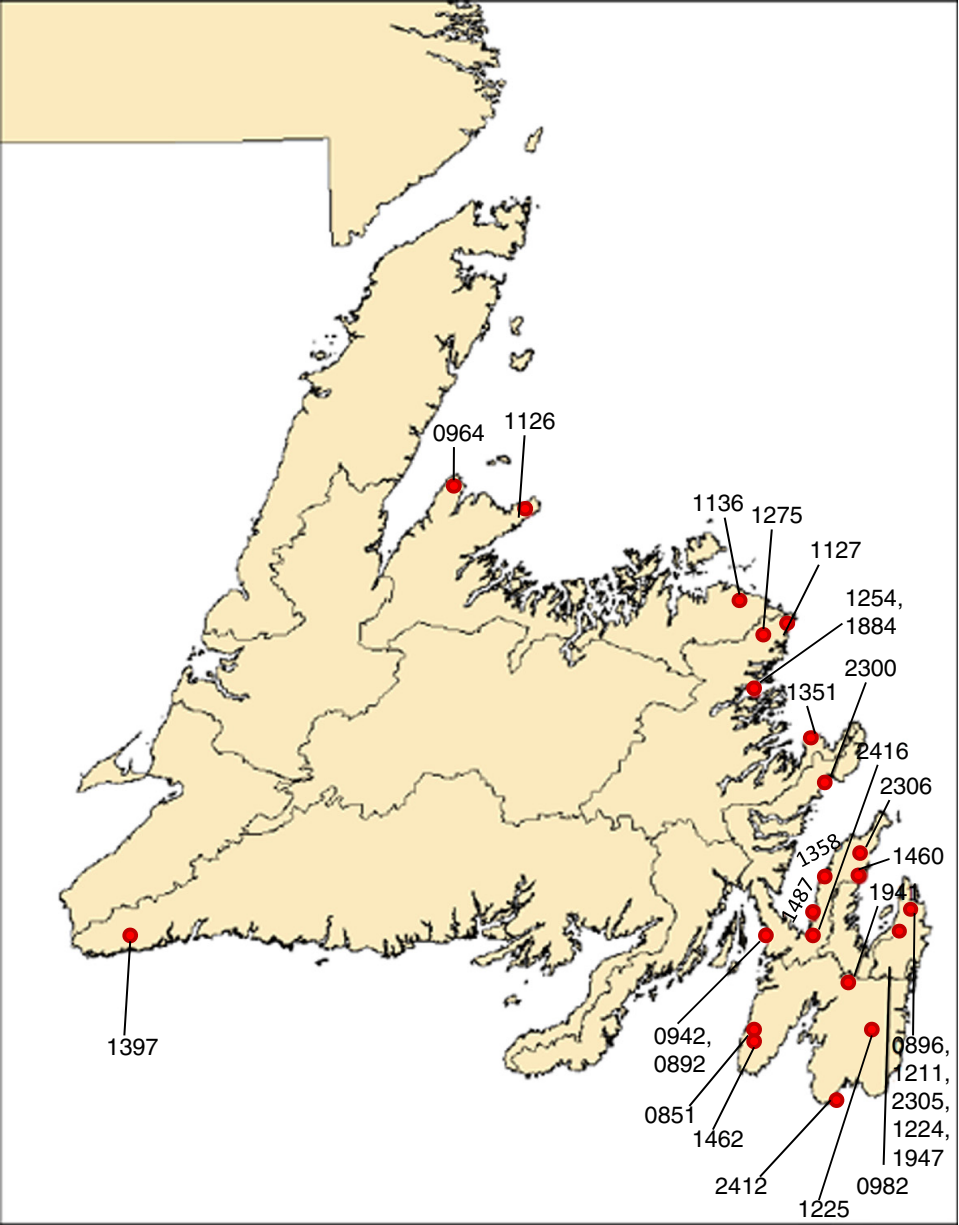
Map of Newfoundland showing geographic distribution of 28 families with familial pulmonary fibrosis.

The only other published IPF cohort from a founder population is Finnish. In comparison to the Newfoundland founder families which are 10–20 generations old, the Finnish population is older, dating back at least 100 generations [33]. Hodgson et al. [9] identified IPF patients in Finland by reviewing hospital databases, and familial cases were ascertained by mailing questionnaires to living IPF patients with a response rate of 56%. Detailed clinical parameters were not published, but the only difference identified between the familial and sporadic groups was again mean age of diagnosis. The seventeen multiplex PF families identified clustered in eastern Finland [9]. The 28 Newfoundland families did not come from a single region of the province (Figure 2), however all families except one originated from either the eastern-most peninsula of the island or from the northeast coast. Nevertheless, we found two distinct *TERT* mutations in two families (R0851, R0892) from the island’s eastern-most peninsula, indicating that neighbouring communities may have independent mutations.

The identified autosomal dominant PF genes account for no more than 15-20% of patients with familial disease [34,35], suggesting that there are unidentified PF genes. Our molecular work supports this hypothesis. Only three probands (all familial) from our total cohort carried a pathogenic variant in a known PF-causing gene (Figure 1). Family R0892 is segregating a *TERT* mutation with five mutation-positive individuals who have developed PF, and six living affected individuals who are mutation-positive without PF. There is also one mutation-positive female who died at 88 years from congestive heart failure, and at age 82, her HRCT scan and PFTs were normal. Apart from premature graying (completely white hair by age 40) in one mutation-positive person with PF, and in 5 mutation-positive individuals without PF, no other telomerase associated medical problems were identified in this family. In R0892, the mean age of diagnosis in the older generation was 72 years compared with age 52.6 years in the younger generation, consistent with the phenomenon of genetic anticipation that has been reported in some families with telomerase mutations ([reviewed in [36]). Individuals with *TERT* mutations theoretically create gametes with relatively short telomeres. If the conceptus is also mutation-positive, this short telomere length cannot be repaired leading to even shorter telomere length for age than was present in the mutation-positive parent. In the second family with a *TERT* mutation (R0851), the three affected brothers in the second generation had other telomerase associated medical problems. All three had bone marrow hypoplasia with chronically low platelets and the older two developed cirrhosis of unknown etiology. The eldest of the three had successful liver and lung transplantations. The three brothers were diagnosed with PF by the second decade of life compared with their mother who was diagnosed at 42 years. In the third *TERT* mutation family (R1254), the father of the proband died of cryptogenic cirrhosis. The father’s chest X-rays showed mild changes consistent with ILD, but no DNA was available to confirm that he was mutation-positive.

One limitation of our study is that we did not analyze telomere lengths in the sporadic patients or in the 25 familial probands who did not have *TERT* mutations. Also although we systematically assessed each participant for liver disease and cytopenias, we did not collect data on premature graying.

## Conclusion

To date, autosomal dominant mutations in four genes are known to cause adult onset IPF: *SFTPC*, *SFTPA2*, *TERC* and *TERT*. We screened these genes in our familial (n = 28) and sporadic (n = 50) probands, 95% of whom belong to the Newfoundland founder population. Except for three familial patients with *TERT* mutations, no causal variants were identified. We hypothesize that at least one novel FPF gene is segregating in this population. We are currently pursuing gene identification through a combination of linkage analyses and exome sequencing.

## Abbreviations

AIP: Acute Interstitial Pneumonia; ATS: American Thoracic Society; COP: Cryptogenic Organizing Pneumonia; CXR: Chest X-Ray; DIP: Desquamative Interstitial Pneumonia; DL_CO_: Diffusing Capacity of the Lung for Carbon Monoxide; ERS: European Respiratory Society; FVC: Forced Vital Capacity; FEV: Forced Expiratory Volume in One Second; FPF: Familial Pulmonary Fibrosis; FPF-DC: Familial Pulmonary Fibrosis - Diagnosed Clinically; FPF-DS: Familial Pulmonary Fibrosis - Diagnosed through Family Screening; HRCT: High Resolution Computed Tomography; IIP: Idiopathic Interstitial Pneumonias; ILD: Interstitial Lung Disease; IPF: Idiopathic Pulmonary Fibrosis; LIP: Lymphocytic Interstitial Pneumonia; NL: Newfoundland and Labrador, Canada; NSIP: Non-Specific Interstitial Pneumonitis; OMIM: Online Mendelian Inheritance in Man; PF: Pulmonary Fibrosis; PFT: Pulmonary Function Test; RB-ILD: Respiratory Bronchiolitis-Associated Interstitial Lung Disease; SD: Standard Deviation; *SFTPC*: Surfactant protein C; *SFTPA2*: Surfactant protein A2; *TERT*: Telomerase reverse transcriptase; *TERC*: Telomerase RNA component; UIP: Usual Interstitial Pneumonitis.

## Competing interests

The authors declare that they have no competing interests.

## Authors’ contributions

BAF is the principle investigator of the study. She designed the study, is the primary author of the manuscript, supervised the phenotypic assessments and takes responsibility for the work as a whole. She contributed to obtaining funding for the manuscript, and to the statistical analyses. GF contributed to the review of the manuscript, to patient recruitment and to the study design. He reviewed all the pulmonary tests and worked with BAF to classify each participant’s status with respect to IPF. RB and ES contributed to the study design and reviewed all the CT scans and chest X-rays. BN was the study’s coordinator. She was responsible for patient recruitment and enrolment, and for the collection of pedigree and other clinical data. She maintained the research database performed geneology searches. NaD and DN reviewed the available pathology specimens for all participants. NiD contributed to patient recruitment. AD, FK and LE contributed to DNA sequencing data collection and interpretation; KM contributed to DNA sequencing, data collection and interpretation, review and preparation of the manuscript; SS performed the statistical analyses of all data. PSP contributed to the study design, obtained funding for the project and reviewed the manuscript. MOW contributed to the writing and final review of the manuscript, to the study design, and to obtaining funding for the manuscript. He supervised all molecular genetic aspects of the project.

## Supplementary Material

Additional file 1 **Table S1.** List of variants in four FPF genes found in affected family members and sporadic patients. Click here for file
