# Supplementary material for: A Newfoundland cohort of familial and sporadic idiopathic pulmonary fibrosis patients: clinical and genetic features
Source: Respir Res. 2012 Aug 1;13(1):64. doi: 10.1186/1465-9921-13-64 (PMC3463483; doi:10.1186/1465-9921-13-64)
Supplement: Additional file 1 — Table S1. List of variants in four FPF genes found in affected family members and sporadic patients. [file 1465-9921-13-64-S1.doc]

**Additional Table 1** **List of variants in four FPF genes found in affected family members and sporadic patients.**

| **Gene** | **Exon/ Intron** | **Variant** | **rs#** | **Predicted protein change** | **SIFT** | **Polyphen** | **Allele frequency** | **European allele frequency** |
| --- | --- | --- | --- | --- | --- | --- | --- | --- |
| **SFTPC** | intron 1 | c.42+72C>A | - | NA | NA | NA | 0.13 (10/76) | NA |
|  | intron 2 | c.43-21T>C | - | NA | NA | NA | 0.33 (19/58) | NA |
|  | exon 4 | c.413C>A | rs4715 | p.Thr138Asn | tolerated | benign | 0.052 (3/58) | 0.23 |
|  | intron 4 | c.435+14G>A | - | NA | NA | NA | 0.069 (4/58) | NA |
|  | intron 4 | c.436-8C>G | rs2070687 | NA | NA | NA | 0.12 (7/58) | 0.24 |
|  | exon 5 | c.557G>A | rs1124 | p.Ser186Asn | tolerated | benign | 0.10 (6/58) | 0.29 |
|  | exon 6 | c.*123G>A | rs7592 | NA | NA | NA | 0.24 (18/76) | 0.30 |
| **SFTPA2** | 5'UTR | c.-120T>G | rs17883188 | NA | NA | NA | 0.93 (106/114) | 0.98 |
|  | 5'UTR | c.-103T>A | rs17881948 | NA | NA | NA | 0.08 (9/114) | 0.06 |
|  | exon 1 | c.-73G>C | rs2271788 | NA | NA | NA | 0.14 (22/160) | 0.86 |
|  | exon 2 | c.-35T>G | - | NA | NA | NA | 0.91 (42/46) | NA |
|  | intron 2 | c.-24+62T>C | - | NA | NA | NA | 0.93 (106/114) | NA |
|  | intron 2 | c.[-23-5G>A];[-23-5G>T] | - | NA | NA | NA | 0.96 (135/140) | NA |
|  | exon 3 | c.26C>A | rs1059046 | p.Thr9Asn | tolerated | possibly damaging | 0.73 (104/142) | 0.82 |
|  | exon 3 | c.56C>T | rs61862677 | p.Ala19Val | tolerated | benign | 0.44 (62/142) | NA |
|  | exon 3 | c.148G>C | rs11554795 | p.Val50Leu | tolerated | benign | 0.19 (6/32) | 0.95 |
|  | exon 4 | c.186G>A | rs2434114 | p.= | NA | NA | 0.78 (86/110) | NA |
|  | exon 4 | c.197C>T | - | p.Thr66Ile | tolerated | benign | 0.89 (98/110) | NA |
|  | exon 4 | c.198A>G | rs1713397 | p.= | NA | NA | 0.89 (98/110) | 1.00 |
|  | exon 4 | c.213G>A | - | p.= | NA | NA | 0.89 (98/110) | NA |
|  | exon 4 | c.217A>G | - | p.Asn73Asp | tolerated | benign | 0.89 (98/110) | NA |
|  | exon 4 | c.241G>A | - | p.Val81Ile | tolerated | benign | 0.85 (94/110) | NA |
|  | exon 4 | c.253C>T | - | p.Arg85Cys | tolerated | benign | 0.86 (95/110) | NA |
|  | exon 4 | c.271G>C | rs17886395 | p.Ala91Pro | tolerated | probably damaging | 0.88 (97/110) | 0.10 |
|  | exon 4 | c.282A>G | rs17886221 | p.= | NA | NA | 0.86 (95/110) | 0.02 |
|  | intron 4 | c.292+11C>T | - | NA | NA | NA | 0.88 (97/110) | NA |
|  | intron 4 | c.292+45G>A | rs17884130 | NA | NA | NA | 0.14 (15/110) | 0.00 |
|  | intron 4 | c.292+46A>C | rs17882071 | NA | NA | NA | 0.14 (15/110) | 0.00 |
|  | intron 4 | c.292+48dupC | - | NA | NA | NA | 0.79 (87/110) | NA |
|  | intron 4 | c.292+64A>G | rs17880662 | NA | NA | NA | 0.73 (80/110) | 0.00 |
|  | intron 4 | c.293-68G>A | rs1610802 | NA | NA | NA | 0.01 (1/112) | NA |
|  | exon 5 | c.342C>T | - | p.= | NA | NA | 0.07 (8/112) | NA |
|  | intron 5 | c.370+67C>G | - | NA | NA | NA | 0.08 (9/112) | NA |
|  | intron 5 | c.370+77T>C | - | NA | NA | NA | 0.02 (2/112) | NA |
|  | intron 5 | c.370+77_78dupTA | - | NA | NA | NA | 0.10 (11/112) | NA |
|  | exon 6 | c.420C>T | rs61862719 | p.= | NA | NA | 0.14 (15/112) | 0.25 |
|  | exon 6 | c.667C>A | rs1965708 | p.Gln223Lys | tolerated | benign | 0.07 (8/112) | 0.11 |
|  | 3'UTR | c.*28G>A | rs17879546 | NA | NA | NA | 0.02 (2/112) | 0.02 |
|  | 3'UTR | c.*29A>G | rs17880428 | NA | NA | NA | 0.01 (1/112) | 0.02 |
|  | 3'UTR | c.*120A>G | - | NA | NA | NA | 0.14 (16/112) | NA |
| **TERT** | exon 1 | c.-42dupC | - | NA | NA | NA | 0.01 (1/112) | NA |
| **†** | exon 2 | c.1321_1323delGAG | - | p.Glu441del | NA | NA | 0.01 (1/112) | NA |
|  | exon 4 | c.1892G>A | - | p.Arg631Gln | damaging | possibly damaging | 0.01 (1/112) | NA |
|  | exon 9 | c.2517G>A | - | p.= | NA | NA | 0.04 (2/46) | NA |
|  | exon 10 | c.2594G>A | rs121918666 | p.Arg865His | damaging | possibly damaging | 0.01 (1/112) | NA |
|  | exon 10 | c.2648T>G | - | p.Phe883Cys | damaging | probably damaging | 0.01 (1/92) | NA |
|  | exon 14 | c.3039C>T | rs33954691 | p.= | NA | NA | 0.11 (5/46) | 0.14 |
| ***** | exon 15 | c.3184G>A | rs35719940 | p.Ala1062Thr | tolerated | benign | 0.01 (1/112) | 0.02 |
|  | exon 16 | c.3324G>A | rs35033501 | p.= | NA | NA | 0.04 (2/46) | 0.04 |
|  | 3'UTR | c.*99C>T | rs2853690 | NA | NA | NA | 0.13 (6/46) | 0.16 |
| **TERC** | 5'UTR | n.-23G>A | - | NA | NA | NA | 0.02 (1/46) | NA |
|  | 5'UTR | n.-11G>A | - | NA | NA | NA | 0.01 (1/108) | NA |
|  | 3'UTR | n.*63A>G | rs2293607 | NA | NA | NA | 0.19 (21/108) | 0.30 |
|  | 3'UTR | n.*111C>G | - | NA | NA | NA | 0.02 (1/46) | NA |

NA=Not Available; SIFT=Sorting Intolerant From Tolerant; p.= indicates there is no amino acid change for this coding variant

**†** This is a rare known variant previously identified in a normal control and shown to be associated with normal telomere length (Yamaguchi H, Calado RT, Ly H, et al. Mutations in *TERT*, the gene for telomerase reverse transcriptase, in aplastic anemia. *N Engl J Med.* 2005;352(14):1413-24).

* This variant was identified as potentially affecting telomerase function in one study (Calado RT, Regal JA, Hills M, et al. Constitutional hypomorphic telomerase mutations in patients with acute myeloid leukemia. *Proc Natl Acad Sci U S A.* 2009;106(4):1187-92) and was also identified in control populations; however, it appears to be more frequent in PF and AML populations than controls. We describe this as an unclassified variant as it is in a sporadic PF patient in our cohort. We also do not know the NL population frequency for the variant nor did we perform telomerase length assays.
